# Effects of Dietary Phytosterol Supplementation on the Productive Performance, Egg Quality, Length of Small Intestine, and Tibia Quality in Aged Laying Hens

**DOI:** 10.3390/ani13040662

**Published:** 2023-02-14

**Authors:** Xiangyu Xiao, Yucheng Zhu, Bohua Deng, Jiaojiao Wang, Shiyi Shi, Shaoshuai Wang, Xiaoqing Han, Ling Zhao, Tongxing Song

**Affiliations:** 1 College of Animal Science and Technology, Huazhong Agricultural University, Wuhan 430070, China; 2 College of Food Science and Technology, Huazhong Agricultural University, Wuhan 430070, China

**Keywords:** phytosterols, aged laying hen, productive performance, egg quality, tibia quality

## Abstract

**Simple Summary:**

A dramatic decline in productive performance and eggshell quality is a common problem for aged laying hens. In recent years, various natural products and medicinal plants have been widely used as alternative nutritional strategies to solve this problem. Phytosterols as natural active ingredients are found in cereal. It is well known that phytosterols have a variety of pharmacological functions. However, the effect of dietary phytosterols on egg quality has been scarcely studied. In the present research, we studied the effects of different concentrations of phytosterols on the productive performance, egg quality, small intestine, and tibia of aged laying hens. The results demonstrated that the supplementation of phytosterols in the diet for aged laying hens improved egg quality, which may be caused by the increase in length of the small intestine.

**Abstract:**

This study aimed at investigating the effects of phytosterols on the productive performance, egg quality, length of small intestine, and tibia quality in aged laying hens. A total of 960 Dawu Jinfeng commercial laying hens (75 weeks of age) were randomly assigned to three groups. Each group had 16 replicates and every replicate contained four cages (five birds/cage). The control group hens received the basal diet without phytosterols. The hens in the experimental groups received a diet containing phytosterols at concentrations of 20 mg/kg and 40 mg/kg for 7 weeks. The results showed that phytosterols had a linearly increasing effect on egg weight, eggshell surface area, albumen height, and haugh unit at week 5 of experiment (*p* < 0.05). Supplemental phytosterols linearly and quadratically increased eggshell thickness (*p* < 0.05). At week 7 of the experiment, dietary supplementation of phytosterols linearly increased egg weight and eggshell weight (*p* < 0.05). Supplementation of 20 mg/kg, but not 40 mg/kg, phytosterols increased the length of the small intestine. However, dietary phytosterols had no effect on the laying rate, mortality, or liver index (*p* > 0.1). The results of tibia quality detected by micro-CT also showed no difference in the treatment of phytosterols. Therefore, supplementation with 20 mg/kg phytosterols in the diet improves egg quality and increases the length of small intestine, but has no effects on the quality of the tibia.

## 1. Introduction

For the egg industry worldwide, the production of eggs with a good external and internal quality is essential for the economic viability of the industry [1]. It is estimated that over 10% of eggs produced in the henhouse are not collected because of egg quality problems, which currently costs the industry many millions of dollars per year [1,2]. In production, many factors can affect the egg quality of hens, especially age [3]. Many studies have shown a correlation between a decline in eggshell quality and advancing age [4,5]. The egg weight, but not eggshell weight, increases with the age of hens. Thus, the increase in egg weight is not accompanied by a proportional change in eggshell weight, which may lead to a reduction in the quality of eggshell [6]. This disproportionate increase will inevitably lead to a reduction in egg quality. It makes modulation of the egg quality more difficult. It is well known that egg quality decreases with the increasing age of laying hens after their laying peak [7,8]. Currently, nutrition regulation has become an important means to improve the persistence and stability of egg quality in poultry.

As a natural and sustainable livestock additive, medicinal plants with active ingredients have shown great potential in recent years [9]. Phytosterols (PS) as natural active ingredients are found in cereal [10]. It is well known that phytosterols have a variety of pharmacological functions such as cholesterol-lowering, anti-inflammatory, and antioxidant properties [11,12,13]. Recently, some researchers have extensively studied the effect of phytosterols in poultry. In broiler production, it has been found that dietary phytosterol supplementation at a dosage of 40 mg/kg can improve white feather broiler growth performance, lipid profile, intestinal morphology, and meat quality [14]. It has also been reported that greater than or equal to 60 mg/kg dietary β-sitosterol reduced the serum total cholesterol and intestinal mucosal malondialdehyde levels; greater than or equal to 80 mg/kg of dietary β-sitosterol effectively improved the intestinal oxidative status, immune function, and morphology; and greater than or equal to 100 mg/kg dietary β-sitosterol increased the length of the ileal villus [15]. In laying hen production, diets enriched with 0, 0.5, 1, and 2% phytosterols had no effect on feed intake, body weight, egg production, liver mass, plasma, and hepatic cholesterol concentrations in single comb white leghorn laying hens [16]. Moreover, it has been reported that a diet enriched with 1% soy sterols also had no significant effect on 28-day weight gain, feed consumption, feed efficiency, plasma total cholesterol, and hen-day egg production in white leghorn hens [17]. Interestingly, as the concentration of phytosterols increased, the eggshell percentage decreased linearly, and the eggshell thickness decreased with a high concentration of phytosterols in Hy-Line Brown hens [18]. Although several studies have investigated the effects of phytosterols on laying hens, the effect of phytosterols on aged laying hens has not been reported. Thus, it is still unclear whether phytosterols improve egg production and egg quality in aged laying hens.

In addition, it has been reported that low quality eggshell will always happen in aged laying hens [19]. The eggshell consists of 97% calcium carbonate, which determines the quality of the eggshell [20]. Therefore, calcium metabolism is one of the main factors affecting the quality of the eggshell. It has been reported that bone quality is closely associated with egg production and egg quality [21]. The main reason for this is that nearly 20–40% of the calcium required for eggshell formation is supplied from bones that are progressively resorbed to provide calcium for eggshell formation [22,23]. Previous studies have also found that calcium is absorbed mostly in the small intestine, accounting for approximately 90% of the overall calcium absorption [24].

Hence, the aim of the present study was to investigate the effect of supplementation with different concentrations of phytosterols on egg production, egg quality, small intestine, and tibia quality in aged laying hens.

## 2. Materials and Methods

### 2.1. Birds and Experimental Design

All of the hens used in this study were humanely managed according to the Chinese Guidelines for Animal Welfare. The experimental procedures were approved by the Institutional Animal Care and Use Committee of Huazhong Agricultural University (HZAUCH-2023-0001). At 75 weeks of age, a total of 960 Dawu Jinfeng commercial laying hens were randomly allocated into three groups. Each group included 16 replicates with four cages (five birds/cage). The control group was fed with a basal diet and the experimental groups received a diet supplemented with different concentrations of phytosterols (20 mg/kg and 40 mg/kg freeze-dried powder of phytosterols). We considered the hens from 75 to 82 weeks of age, and the total experimental period was 7 weeks. The hens were monitored for 1 week prior to the start of the experiment (baseline period). During this period, the hens were fed the basal diet and the productive performance was similar between groups. All of the hens were housed in a room that was maintained at 22 °C throughout the experiment and the light/dark program was 16 h/8 h. All of the hens were allowed access to water and feed ad libitum.

### 2.2. Experimental Diet and Groups

According to NRC (1994) [25], the basal diets were prepared according to a corn–soybean-type diet. The ingredients and chemical composition of the basal diets are listed in Table 1. The control group was fed a basal diet only, and the phytosterol groups were fed a basal diet that contained phytosterols. The phytosterol groups consisted of a basal diet supplemented with 20 mg/kg or 40 mg/kg phytosterols. The form of phytosterols was freeze-dried powder and was uniformly mixed into the basal diet. The content of phytosterols was 96%, purchased from Nanjing Nature Bio-Tech Co., Ltd., Nanjing, China.

### 2.3. Sample Collection and Analytical Determination

During the experiment, all of the eggs and dead hens were collected and recorded daily in all replicates throughout the experimental period. The egg production was expressed as the average daily production.

#### 2.3.1. Sample Collection in Week 5

Five eggs were randomly collected from each replicate and a total of 80 eggs (5 eggs × 16 replicates) per group were used for the interior and exterior quality tests at the end of 5 weeks.

#### 2.3.2. Sample Collection in Week 7

At the end of the feeding trial, 12 replicates were chosen randomly from 16 replicates per group. Five eggs were randomly selected from these 12 replicates and a total of 60 eggs (5 eggs × 12 replicates) per group were used for the external quality tests. One hen from each replicate was randomly selected. A total of 12 hens (1 hens × 12 replicates) per group were weighed, euthanized by manual cervical dislocation, and exsanguinated via the left jugular vein [26]. After exsanguination, the liver, small intestine, and tibia were collected.

#### 2.3.3. Sample Analytical Determination

##### Tibia Quality

The lengths of the tibias were measured. The tibias were scanned using a micro-computed tomography scanner (Skyscan 1276 Mi-cro-CT, manufactured by Bruker Magnetic Resonance, Germany) and analyzed by the NRecon and CTan integrated software. Bone mineral density (BMD) and bone mineral content (BMC) were analyzed from this software.

##### Intestine and Liver

The length of the small intestine and the weight of the liver were measured. The liver index used the following formula: organ index (%) = organ weight/body weight × 100.

##### Egg Quality

Egg quality refers to various standards of the external and internal quality of an egg. The internal quality of the egg typically includes the yolk color, albumen height, and haugh unit. Similarly, the external quality contains the eggshell thickness, eggshell strength, and eggshell surface area. The eggshell surface area was calculated using the following formula: eggshell surface area = 4.68 × (egg weight)^2/3^ [27]. Eggshell strength was evaluated using an eggshell force gauge (Tenovo KQ-1A, Tenovo International Co., Ltd., Beijing, China). Egg weight, egg yolk color, albumen height, and haugh unit were evaluated using an egg multitester (EMT-7,300 II, Robotmation Co., Ltd., Tokyo, Japan). The eggshell was gently washed and air dried to remove the shell membrane, and then weighed. A 200-mm digital vernier caliper was used to measure the sharp, equator, and blunt of the egg. The average of three thickness values from the sharp, equator, and blunt of the egg shell was calculated. The values of the egg quality for each replicate were the average of five eggs.

### 2.4. Statistical Analyses

All data analyses were conducted using the IBM SPSS statistical package (Version: SPSS 25.0, IBM Corp, Armonk, NY, USA). One-way ANOVA was used for the significance analysis of the productive performance, egg quality, length of the small intestine, length of the tibia, and BMC among the different groups. Levene’s test for homogeneity of variance was performed and Welch’s ANOVA was used when the variances were unequal. Combined with the LSD method, multiple comparisons were performed, with *p* < 0.05 being of statistical significance. The results were expressed as mean ± SEM. Because the body weight, liver weight, eggshell thickness, and BMD were not normally distributed, a Kruskal–Wallis one-way nonparametric ANOVA was used to determine the overall differences among the groups. The mortality was analyzed by chi-square. Orthogonal polynomial contrasts were used to test the linear and quadratic effects of the increasing levels of dietary phytosterols.

## 3. Results

### 3.1. Productive Performance

The effect of phytosterols on the productive performance is presented in Table 2. From 1 to 7 weeks, dietary supplementation of graded levels of phytosterols had no effect on egg production (*p* > 0.05). Moreover, there was no relationship between the level of dietary phytosterols and mortality throughout the experimental period (*p* > 0.05).

### 3.2. Egg Quality

At week 5 of the experiment, as described in Table 3, the inclusion of phytosterols in the diet of laying hens had a linear effect through increasing the egg weight and eggshell surface area (*p* < 0.05). Moreover, supplemental phytosterols linearly and quadratically increased the eggshell thickness (*p* < 0.05). For the internal quality of the egg, phytosterols in the diet had a linear effect through increasing the albumen height and haugh unit (*p* < 0.05), and linearly and quadratically decreased the yolk color (*p* < 0.05). At the end of the experiment, the egg weight showed a linear increase in response to the increased dietary phytosterol levels (*p* < 0.05), and the dietary supplementation of different levels of phytosterols linearly increased the eggshell weight (*p* < 0.05). However, phytosterol supplementation had no significant effect on eggshell thickness, eggshell strength, and eggshell percentage (*p* > 0.1).

### 3.3. Tibia Quality and Intestine and Liver Characteristics

**Figure 1:**
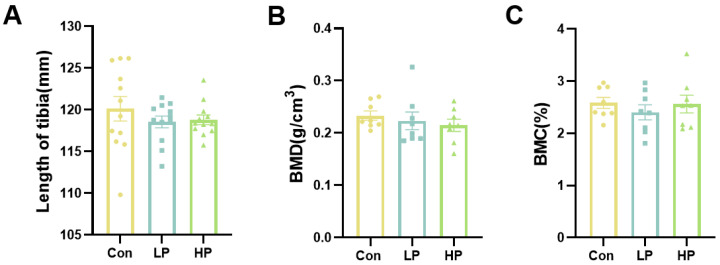
Effects of dietary phytosterols on tibia quality in aged laying hens. (**A**) Length of tibia, *n* = 12; (**B**) bone mineral density (BMD), *n* = 8; (**C**) bone mineral content (BMC), *n* = 8. Data are presented as mean ± SEM.

To investigate whether the changes in egg quality were associated with the mobilization of calcium from the bone, the tibia quality was analyzed. The effects of phytosterols on tibia quality are presented in Figure 1. The results show that phytosterols had no significant effects on the length of the tibia, weight of tibia, bone mineral content (BMC), or bone mineral density (BMD). As shown in Table 4, the results revealed that supplemental phytosterols at 20 mg/kg, but not at 40 mg/kg, increased the length of the small intestine (*p* < 0.05). Meanwhile, dietary phytosterol administration had no significant effects on body weight, liver weight, and liver index at the end of the experiment (*p* > 0.05, Table 4).

## 4. Discussion

Our results found that dietary phytosterol supplementation has no effect on productive performance, which is also consistent with previous studies [16,17,28]. These studies show that feeding phytosterols to laying hens had no significant effect on increasing egg production. Moreover, feeding phytosterols to aged laying hens had no significant effect on the mortality or liver index.

The decrease in egg quality in aged laying hens is well known in poultry production. In our study, some improvements were found in the external quality of the egg (egg weight, eggshell surface area, and eggshell thickness), but not in the eggshell strength in aged laying hens after phytosterol treatment for 5 weeks. Moreover, the internal quality of the eggs, including the albumen height and haugh unit, was also improved. Notably, the administration of phytosterols for 7 weeks mainly increased the egg weight. The possible reason for the inconsistency between the two results is the different duration of feeding phytosterols. However, the exact mechanism of how phytosterols modulate egg quality is still unknown. Therefore, further research will be needed to explore the effect of phytosterols on the egg quality parameters, particularly regarding the mechanism of improving egg quality.

In addition, it has been reported that egg quality is closely associated with bone quality [21]. Therefore, the quality of bones can also reflect the quality of eggs. Previously, it has been shown that the combination of phytosterols and β-cryptoxanthin reduces the risk of osteoporosis in postmenopausal women [29]. This suggests that phytosterols have the effect of regulating bone metabolism. However, our results have shown that dietary phytosterol supplementation has no effect on the length of the tibia, weight of the tibia, BMC, or BMD. Given the inconsistent effect of the duration of feeding phytosterols on egg quality found in this study, we speculate that the reason the addition of phytosterols to the diets has no significant effect on the bones of aged laying hens is also due to the duration of feeding phytosterols.

The calcium of egg is mainly absorbed through the intestine, and the rest comes from the mobilization of the bone [30]. Previous studies have found that calcium is absorbed mostly in the small intestine, accounting for approximately 90% of the overall calcium absorption [24]. We found that dietary phytosterol supplementation with 20 mg/kg increased the length of the small intestine. Given that phytosterols had no effect on the tibia quality, but enhanced the length of the small intestine in this study, we speculate that 20 mg/kg phytosterols may improve the small intestinal calcium metabolism of aged laying hens so as to improve egg quality, which deserves further exploration for the potential mechanism.

## 5. Conclusions

In summary, the present study indicated that some improvements were found in the external quality of the egg (egg weight, eggshell surface area, and eggshell thickness), but not in the eggshell strength in the aged laying hens after phytosterols treatment for 5 weeks. Moreover, the internal quality of the egg including the albumen height and haugh unit were improved. Moreover, the length of small intestine was also increased by feeding 20 mg/kg, but not 40 mg/kg, phytosterols to the aged laying hens. This research has practical significance for the application of phytosterols in aged laying hens. The mechanism how the changes in the small intestine caused by phytosterols affect egg quality is worthy of further exploration. Further studies are required to clarify the mechanism of dietary phytosterols on egg quality in aged laying hens.

## Figures and Tables

**Table 1:** Composition and nutrient level of the basal diet.

Ingredients	Contents, %
Corn	52.2
Wheat Bran	6.00
Soybean meal	26.1
Soybean oil	4.00
Dicalcium phosphate	1.53
Limestone	8.76
Salt	0.30
Methionine	0.11
Premix ^1^	1.00
Total	100
Calculated nutrient level	
Metabolizable energy MJ·kg^−1^	11.45
Crude protein	16.66
Calcium	3.51
Total phosphorus	0.61
Available phosphorus	0.38
Lysine	0.85
Methionine	0.35

^1^ Provided the following (per kg of diet): vitamin A (trans-retinyl acetate), 12,000 IU; vitamin D3 (cholecalciferol), 4000 IU; vitamin E (dl-α-tocopheryl acetate), 35 IU; vitamin K (bisulfate menadione complex), 5 mg; thiamine (thiamine mononitrate), 2 mg; riboflavin, 8 mg; pyridoxine (pyridoxine HCl), 5 mg; vitamin B12 (cyanocobalamin), 50 μg; D-biotin, 200 μg; pantothenic acid (D-calcium pantothenate), 15 mg; nicotinic acid, 50 mg; choline (choline chloride), 500 mg; folic acid,1.5 mg; Mn, (MnSO_4_, H_2_O), 120 mg; Zn (ZnO), 80 mg; Fe (FeSO_4_, H_2_O), 120 mg; Cu (CuSO_4_·5H_2_O), 15 mg; I (KI), 1 mg and Se (Na_2_SeO_3_), 0.3 mg.

**Table 2:** Effects of dietary phytosterols on the productive performance in aged laying hens.

Items	Dietary Phytosterols Level (mg/kg)			SEM ^1^	*p*-Value			χ^2^	*p*-Value
	0	20	40		PS	Linear	Quadratic		
Egg production ^2^, %									
1 wk	87.760	89.306	90.414	0.013	0.393	0.176	0.896	-	-
2 wk	87.835	87.503	87.445	0.012	0.970	0.819	0.926	-	-
3 wk	75.714	72.190	70.774	0.029	0.476	0.239	0.770		
4 wk	86.935	85.009	87.570	0.016	0.471	0.787	0.274		
5 wk	90.918	89.784	91.457	0.015	0.739	0.807	0.463		
6 wk	90.418	90.261	91.848	0.016	0.753	0.540	0.666		
7 wk	88.530	88.432	91.303	0.016	0.352	0.220	0.446		
1–2 wk									
Survival	320	319	317	-	-	-	-	3.515	0.173
death	0	1	3	-	-	-	-		
All hens	320	320	320	-	-	-	-		
Mortality, %	0	0.312	0.938						
6–7 wk									
Survival	317	319	316	-	-	-	-	3.490	0.175
death	3	0	1	-	-	-	-		
All hens	320	319	317	-	-	-	-		
Mortality, %	0.938	0	0.315						

^1^ SEM, standard error of mean. ^2^ results are means with *n* = 16 per group.

**Table 3:** Effects of dietary phytosterols on egg quality in aged laying hens.

Items	Dietary Phytosterols Level (mg/kg)			SEM ^1^	*p*-Value		
	0	20	40		PS	Linear	Quadratic
5 wk ^2^							
Egg weight, g	60.263 ^a^	63.013 ^b^	64.112 ^b^	0.542	<0.001	<0.001	0.224
Eggshell thickness, mm	0.299 ^a^	0.344 ^b^	0.339 ^b^	0.005	<0.001	<0.001	0.001
Eggshell strength, N	37.908	36.103	34.728	0.971	0.085	0.028	0.860
Eggshell surface area ^3^, cm^2^	74.695 ^a^	76.949 ^b^	77.839 ^b^	0.442	<0.001	<0.001	0.217
Albumen height, mm	6.061 ^a^	6.813 ^b^	7.182 ^b^	0.238	0.007	0.002	0.523
Haugh unit	75.125 ^a^	79.073 ^ab^	81.204 ^b^	1.592	0.034	0.011	0.649
Yolk color	11.880 ^a^	11.011 ^b^	11.151 ^b^	0.093	<0.001	<0.001	<0.001
7 wk ^4^							
Egg weight, g	60.730 ^a^	63.387 ^b^	63.340 ^b^	0.475	<0.001	<0.001	0.027
Eggshell thickness, mm	0.329	0.331	0.332	0.003	0.961	0.519	0.880
Eggshell strength, N	40.352	39.272	39.043	1.111	0.686	0.420	0.761
Eggshell weight, g	5.670 ^a^	5.836 ^a^	5.954 ^b^	0.059	0.007	0.002	0.746
Eggshell percentage ^5^, %	9.344	9.213	9.403	0.119	0.528	0.732	0.284

Note: In the same line, values with different letters are significantly different (*p* < 0.05). ^1^ SEM, standard error of mean. ^2^ results are means with *n* = 16 per group. ^3^ Eggshell surface area: 4.68 × (egg weight)^2/3^. ^4^ results are means with *n* = 12 per group. ^5^ Eggshell percentage: eggshell weight/egg weigh.

**Table 4:** Effects of dietary phytosterols on the length of small intestine and the weight of liver in aged laying hens ^1^.

Items	Dietary Phytosterols Level (mg/kg)			SEM ^2^	*p*-Value		
	0	20	40		PS	Linear	Quadratic
Body weight, kg	1.710	1.737	1.834	0.067	0.662	0.204	0.673
Liver weight, g	40.044	37.894	40.403	2.427	0.854	0.921	0.459
Liver index, %	23.519	22.033	21.956	1.134	0.565	0.346	0.622
Length of small intestine, mm	115.292 ^a^	133.125 ^b^	109.440 ^a^	4.304	0.001	0.355	0.001

Note: In the same line, the values with different letters are significantly different (*p* < 0.05). ^1^ results are means with *n* = 12 per group. ^2^ SEM, standard error of mean.

## Data Availability

The data presented in this study are available upon request from the corresponding author.
